# Industrial Independence

**Published:** 1914-11

**Authors:** 


**A Monthly Review of Medicine and Surgery**

EDITOR AND PUBLISHER, DR. A. L. BENEDICT, 228 Summer St., corner of Elmwood Ave., Buffalo, (Address for all communications. Please make personal and telephone calls before 1 P. M.)

Third, in age, on the western continent. Independent, but supports professional organization. News limited mainly to Western N. Y. and Penn.

Subscription $2.00 a year; $3.00 for two years in advance. Changes and errors in address or failure or duplication of delivery, should be reported immediately.

Yearly Volume 70. NOVEMBER, 1914 No. 4

### Industrial Independence.

While the war of flesh and blood is raging in Europe, this country is forcibly reminded that it must begin an industrial war of independence. The American Tariff League publishes in the American Economist of September 4, a long list of articles for which we are more or less dependent upon Europe. For some of these, as amber, asphalt, chamois skins (genuine), olive oil, and some minerals concerning whose presence in the U. S. in sufficient abundance, we can not speak with certainty, there is ample reason for dependence. But why should aniline dyes, cyanids, egg albumin, hemp, potato starch, salt, starch, sugar, talc, watch crystals, wind shields, wool and zinc be imported? A medical journal is no place for politics but we may voice the neutral opinion that, on the one hand, native production and manufacture should be favored in its incipience either directly by some form of subsidy or indirectly by a protective tariff and, on the other hand, that neither method of artificial support should be continued indefinitely to the extent of stultfying ingenuity and economy of production and manufacture, nor of establishing the principle that our people will not earn their daily bread by honest sweat of body and brain but must have five-course dinners as the reward of very gentle exercise.

Some few of the products mentioned are of interest to the medical profession: rubber blankets, magnesia, tungsten, vanadium, carbolic acid, washed clay, cotton, etc., but, for the most part, actual drugs are omitted. Unfortunately, many vegetable drugs either do not or cannot grow in our climates or, as has been demonstrated in the case of cinchona, may grow thriftily but do not yield an adequate amount of active principle. We are optimistic enough to believe that all but a very small list of mineral products may be mined and refined in our own country. A good many medicinal plants including some of the first rank, are either indigenous to our country or may be cultivated here. Our national and state governments and some of our private educational institutions should recognize that the time is ripe for placing medical botany and horticulture on both a scientific and a practical, industrial basis. The rudiments of such courses involve no new principles or knowledge. Botanies half a century old and older contain fairly accurate notes of the medicinal uses of plants and the botanic school of medicine flourished in the time of our grandfathers. To a considerable degree, the value of vegetable drugs has been exaggerated but, as we have said elsewhere, a drug may be of slight physiologic potency and yet of considerable value therapeutically, in proper cases, and it may be rarely indicated and yet be very much needed when it is indicated at all. The Leonard Drug Co. of Bradford, Pa., gave the writer a pamphlet entitled Wealth in Weeds, on the occasion of a recent visit to that city. This lists some sixty common plants, with the part used and the price per pound, pictures of many plants, and gives directions for collecting. As closely as we can approximate the practical value in therapeutics of these drugs, about a third are almost indispensable to the general practitioner and at least half a dozen could be used without apology to a pharmacologist or indeed to any but a therapeutic nihilist. Studies along the same general line have been published by the government and, fragmentarily by various authors.

But, under present conditions, we feel that the subject should be taken up along much broader lines, so as to include the raising of foreign plants under suitable conditions, and with such financial backing from some of our larger philanthropies or from the government, national or state, as may carry the matter from the stage of mere knowledge, more or less inaccessible to the ordinary student to that of a definitely developed branch of education, analogous to forestry, economic geology and entomology, as applied in the conservation of plant life and in sanitation. Furthermore, actual industrial utilization of this knowledge should be effected.

Fortunately or unfortunately, according to the point of view, many of the plants classifiable as medicinal, are not in great demand, and are scarcely susceptible of cultivation to the exclusion of ordinary crops, pasturage, etc., either because of the low price of the product or because of the conditions necessary to growth. But, for children, certain types of invalids, and for vacation work, the collection of even wild weeds combines a moderate financial reward with opportunities for study and recreation.

As we have previously stated, there is almost nothing in the line of therapeutic products of animal origin or of what may be termed biologic products used in medicine, which cannot be as well prepared in this country as in Europe.

Neither do we believe that, in availability, equipment and quality of manufacturers of therapeutic products, our country need be dependent upon any foreign nation.

We anticipate further, that we are almost at the point of chemic development at which not only ordinary compounds but peculiar allotropic forms of certain elements and compounds and the whole range of organic chemistry will be entirely independent of anything but an available source of raw material of the simplest kinds. With very few if any exceptions, which certainly should include none but the rarest elements, our country has this raw material. It is with a sense of shame that we admit that carbolic acid, gallic acid and aniline derivatives are imported. The time is ripe to consider the subject of synthetic drugs, not from the ethical standpoint nor from the commercial aspect derived from patents, but from one of economic independence.

We cannot neglect the medical phase of manufactured articles. With the possible exception of some few scientific instruments, there is almost nothing either in the way of diagnostic instruments, surgical and electrical instruments, office, hospital and sick room equipment, that cannot be made just as well in this country as in any other. To take a particular instance, rubber is historically an American development. As we ordinarily use the word, we almost forget its meaning, but, except for American inventive genius, it would be of no greater use than for rubbing out pencil marks. Even for this original, trivial use, that same genius has devised better means. We erase by striking an x through a mis-struck type letter or, if we still write by hand, we confess the error by running a line through it, to save time. And, for such few erasures by friction as are needed, we have a better substance in artificially prepared cellulose. It is a disgrace to depend for any rubber fabric or implement upon foreign countries, except for the raw material and for a large part of this we are the logical purchasers and manufacturers. We tread on dangerous ground in claiming that for all but a few manufactured articles used by the medical profession, either as scientific or practical instruments and equipment, our dependence upon a foreign market can be explained by the single illustration of the applicator. The applicator is a very useful instrument but not one representative of a great degree of complexity or professional esteem. But it is a medical instrument and, until a very few years ago, it sold for a dollar. If we analyse it, it is nothing but a short piece of nickle or silver plated wire stuck in a piece of hard rubber. Any comparable implement sold to a dress maker or a carpenter, would cost about ten cents. If we compare the prices of the old fashioned piston pump for laryngologists with essentially the same outfit for pumping up tires, or the spray cautery with a burned wood outfit, or if we visit a high-grade hardware store and compare analogous tools with our instruments, we find the same comparative schedule of prices. There are about 125,000 physicians in the country, wearing out their equipment or discarding it for improved articles with a rapidity that does away with any argument of high cost of production for a limited market. The plain fact is that our country has not developed this market as it has analogous ones and, to a large degree, it has either imported its medical instruments or their makers. We do not wish to be understood as advocating poorly made instruments or as failing to allow for obvious differences in quality and workmanship of professional instruments as compared with trade tools but we do insist that, in spite of every effort to protect American manufacturers even to the extent of allowing them free importation of workmen, we have failed to develope industrial independence of Europe, largely on account of ignoring actual values of labor and material, a factor in competition that nothing short of a Chinese Wall can nullify.

